# Supplementary material for: Balance control mechanisms do not benefit from successive stimulation of different sensory systems
Source: PLoS One. 2019 Dec 11;14(12):e0226216. doi: 10.1371/journal.pone.0226216 (PMC6905548; doi:10.1371/journal.pone.0226216)
Supplement: Attachment — Submitted filename: Response2Reviewer.docx [file pone.0226216.s001.docx]

November 15^th^, 2019

Dear Editor,

We would like to thank you, as well as your associate editor and reviewers for your time and thoughtful critique of our manuscript.

We have considered and reviewed each comment carefully, and our responses can be found directly beneath the original reviewer’s comment.

**Reviewer 1 comment #1:**

AP force: what is the rational for this measure. It is assumed that if there is a sustained shift in the sway of the body (forwards, backwards) then the AP GRF is necessary to prevent a fall. This may not be clear to all readers. Therefore, it is recommended that 1-2 sentences be included to better explain what the AP force represents.

**Authors reply to comment #1:**

As suggested by the reviewer, we added few sentences to clarify the relationship between AP GRF and shift in the AP sway of the body. This can be found in the Methods section of the revised version of the article.

**Reviewer 1 comment #2:**

Time after stimulus: there is litterature that suggests that more than 15 seconds of data is required to obtain stable measure of stability (CoP data). Although the study demonstrates that re-stabilization occurrs within a shorter period of time, it does present an opportunity to comment on this if judged relevant by the authors. See the manuscript by Carpenter et al. about minimum time required to obtain stable sample of COP data.

**Authors reply to comment #2:**

We thank the Reviewer for pointing out this reference from Carpenter et al., (2001). We agree with the conclusion of this manuscript. However, in Carpenter et al.’s manuscript, the authors determined how long the sampling duration had to be before observing no more change in the COP measures in absence of alteration in sensory cues. In contrast, in our study, we were interested in assessing the sensorimotor control mechanisms immediately following a sudden change in sensory state. The values for the stabilization time calculated using our algorithm are like the results reported by Asslander and Peterka (2016), as well as Honeine and Schieppati (2014).

In their manuscript Carpenter et al.’s, assessed how long the sampling duration had to be to observe stable COP measures while in our manuscript, we were interested in determining how long it took to observe a baseline like value of the COP-COG scalar distance. Overall, we think that discussing the difference between our measure of stabilization time and Carpenter et al.’s conclusion would confuse the readers.

Thank you for considering the revised version of our manuscript,

Sincerely,

Martin Simoneau, PhD

Professor, Laval University, Faculty of Medicine – Department of kinesiology

Researcher, Centre for integrative research and social integration (CIRRIS)

Quebec, Qc, Canada
